# Primary insomnia treated with Zolpidem in an 18-month-old child

**DOI:** 10.4103/0019-5545.39763

**Published:** 2008

**Authors:** Tushar Bhat, Sheryl John Pallikaleth, Nilesh Shah

**Affiliations:** Department of Psychiatry, JMFs ACPM Medical College, Dhule, Maharashtra, India

**Keywords:** Children, primary insomnia, Zolpidem

## Abstract

This report describes a case of an 18-month-old child who was treated for primary insomnia with Zolpidem. To the best of our knowledge, the published literature is devoid of any information on use of Zolpidem in infants and children.

## INTRODUCTION

The American Psychiatric Association's Diagnostic and Statistical Manual of Mental Disorders (DSM-IV) defines insomnia as a complaint regarding the quantity, quality, or sleep timing at least three times a week for at least 1 month. In a community survey, Richman found that 20% of normal 1- to 2-year-olds had sleep problems.[1] Mindell suggests that about 25% of children between the ages of 1 year and 5 years experience sleep disturbances.[2] Bedtime resistance has been described as one of the most prevalent sleep problems (27%). Sleep-onset delays (11.3%), night waking (6.5%), morning wake-up problems (17%), and fatigue complaints (17%) were also common in children. Among children with sleep-onset problems, 80% displayed bedtime resistance, while 34% of bedtime resisters had onset problems.[3]

A growing body of evidence shows that childhood sleep disturbances may have a wide-ranging impact on children's health; behavior; mood; neurobehavioral parameters such as attention, cognition, and memory; and school performance; as well as on parental stress and family life. A number of empirically sound and effective behavioral and cognitive/behavioral approaches to the treatment of pediatric insomnia, defined as significant and persistent difficulty in initiating and/or maintaining sleep, have been developed.[4–6]

Although most sleep disturbances in children are managed with behavior therapy alone, pharmacologic intervention or a combination of behavioral and pharmacologic interventions has also been used by both parents and practitioners to treat symptoms of insomnia in children and adolescents. A wide variety of medications have been prescribed or recommended by pediatric practitioners for sleep disturbances in children, including antihistamines, chloral hydrate, barbiturates, phenothiazines, tricyclic antidepressants, benzodiazepines, and α-agonists, in addition to over-the-counter medications containing opium and herbal preparations.

Zolpidem, an imidazopyridine, is a nonbenzodiazepine hypnotic indicated for the short-term treatment of insomnia in adults. In the clinical trials that investigated the use of a hypnosedative drugs in an “as-needed” regimen, Zolpidem produced global improvement in sleep. Though Zolpidem continues to be a useful therapeutic option in the pharmacological treatment of adult patients with insomnia,[7–9] there is little information on use of this drug in children. This report describes a case of an 18-month-old child who was treated for primary insomnia with Zolpidem. To the best of our knowledge, the published literature is devoid of any information on use of Zolpidem in infants and children.

## CASE REPORT

An 18-month-old male child was brought by his parents with a complaint of less sleep, of only 2-5 h/day since birth. According to his mother, who is working as a staff nurse at a hospital, he was born of a full-term normal delivery and there were no perinatal complications at the time of his birth. Soon after his birth, it was noticed by his parents that unlike other newborns who sleep for almost 18-20 h/day, he would sleep only for about 1-2 h/day. He would be awake almost the whole day; and even after breast-feeds or patting him, he would sleep only for a few minutes at a time. He would be awake within a short while and seek his parent's attention by crying. Even at night, in spite of all their efforts, he would not sleep for more than a few minutes at a time and so one of the parents would have to stay awake the whole night with him. Over a period of next 5-6 months, though his sleep improved, he would not sleep for more than 2-3 h at night (30-45 min at stretch, three to four times per night) and 1-2 h during daytime. Although he slept only for 2-5 h everyday, he would be active and cheerful throughout the day and his developmental milestones were not delayed. Subsequently for this problem, a pediatric consultation was sought and he underwent various investigations, including a barium meal, with follow-up to rule out abdominal colic as a cause of persistent insomnia. When all the investigations carried out over a period of next 1 year turned out to be normal and still his problem persisted, he was referred for psychiatric consultation. On psychiatric evaluation, it was observed that he was quite active, cheerful; and initiated and maintained eye-to-eye contact. He exchanged toys and played with them appropriately. He imitated the doctor's behavior of talking on phone by saying “hello, hello.”. No emotional or behavioral problems were observed in him.

In view of this history of persistent insomnia for the last 18 months (since birth) in the absence of any emotional or behavior problems, he was diagnosed as a case of primary insomnia. After discussion about his diagnosis and treatment with his parents and obtaining verbal informed consent, he was started on tablet Zolpidem (extended-release preparation) one-fourth of a 6.25-mg tablet at bedtime (i.e., about 1.5 mg/day). On a follow-up visit after a week, his parents gladly reported a remarkable improvement in his sleep. According to his mother, within few minutes of giving Zolpidem at bedtime, he would fall asleep and would remain fast asleep for 8-10 h at a stretch throughout the night. He started sleeping for 2-3 h at a stretch in the afternoon as well. No drowsiness or hangover was noticed when he woke up in the morning, and he would be as active and cheerful as before. Parents were particularly happy; as now, even they could sleep continuously at night for 8 h without frequent awakenings.

The treatment was continued for a month; and as he was sleeping well, the parents discontinued the medication. In spite of discontinuation of medication, it was observed by the patents that he continued to sleep for about 6 h at night, with which they were happy. This lasted for about 3-4 months, when again parents sought our consultation as again he started sleeping less, only for a couple of hours at night. The parents were asked to consider restarting the medication. Subsequently they were lost to follow-up.

## DISCUSSION

Sleep disorders in infants and children may not be uncommon, but safety and efficacy of any of the sedative-hypnotic drugs have not been established in children. Kurta *et al.* have reported a case series of unintentional consumption of Zolpidem by children, in which there were no major serious adverse events or death reports.[10] In this kind of situations, the question is whether to consider this as a normal variation and leave it alone, hoping for a spontaneous remission, or to consider it as a sleep disorder and treat it. In case of this patient, as insomnia had persisted for 18 months and parents requested treatment, Zolpidem was considered; and on follow-up visit, a remarkable improvement in his sleep was noted. It is too premature to claim that Zolpidem is safe and effective based on a single case study. This report should not be construed as evidence that Zolpidem can be used in children, and more systematic studies are needed to verify the findings of this case.
